# Comparative Degradation of a Thiazole Pollutant by an Advanced Oxidation Process and an Enzymatic Approach

**DOI:** 10.3390/biom7030064

**Published:** 2017-08-24

**Authors:** Khadega A. Al-Maqdi, Soleiman M. Hisaindee, Muhammad A. Rauf, Syed Salman Ashraf

**Affiliations:** Department of Chemistry, UAE University, P.O. Box 15551, Al-Ain, UAE; 200935138@uaeu.ac.ae (K.A.A.-M.); soleiman.hisaindee@uaeu.ac.ae (S.M.H.); Muhammada@uaeu.ac.ae (M.A.R.)

**Keywords:** bioremediation, thiazole, advanced oxidation process, peroxidases, enzymes, chloroperoxidase

## Abstract

Organic pollutants, especially those found in water bodies, pose a direct threat to various aquatic organisms as well as humans. A variety of different remediation approaches, including chemical and biological methods, have been developed for the degradation of various organic pollutants. However, comparative mechanistic studies of pollutant degradation by these different systems are almost non-existent. In this study, the degradation of a model thiazole pollutant, thioflavin T (ThT), was carried out in the presence of either an advanced oxidation process (ultraviolet (UV) + H_2_O_2_) or a chloroperoxidase enzyme system (CPO + H_2_O_2_). The degradation was followed both spectrophotometrically and using liquid chromatography-mass spectroscopy (LC-MS), and the products formed were identified using tandem liquid chromatography-mass spectrometry-mass spectrometry (LC-MS-MS). The results show that the two remediation approaches produced different sets of intermediates, with only one common species (a demethylated form of ThT). This suggests that different degradation schemes were operating in the two systems. Interestingly, one of the major intermediates produced by the CPO + H_2_O_2_ system was a chlorinated form of thioflavin. Phytotoxicity studies showed that the CPO + H_2_O_2_-treated ThT solution was significantly (*p* < 0.05) less toxic than the UV + H_2_O_2_-treated ThT solution. This is the first time that a comparative mechanistic study showing in detail the intermediates generated in chemical and biological remediation methods has been presented. Furthermore, the results show that different remediation systems have very different degradation schemes and result in products having different toxicities.

## 1. Introduction

Aromatic compounds are a major class of toxic and potentially carcinogenic organic pollutants that must be removed from effluents before they enter bodies of water [1,2,3]. Various physical, chemical, and biological approaches have been devised to deal with these contaminants. For example, adsorption, sedimentation, coagulation, membrane filtration, and use of cucurbiturils are all examples of physical processes that have been successfully used to remove various organic pollutants [4,5]. Some of the most commonly used chemical methods include ozonation, NaOCl treatment, and advanced oxidation processes (AOPs) [6,7].

Although physical and chemical methods enjoy wide-scale applicability and are currently used in various large-scale processes, they still face some significant limitations and challenges. The biggest downside of physico-chemical methods are the high costs involved as well as the sludge produced by the processes [4]. Greener alternatives, such as the use of micro-organisms or phytoremediation, are considered to be more environmentally friendly and have shown promising results for the removal of low concentrations of organic pollutants. Compared with traditional physico-chemical methods, bioremediation may be a safer, less disruptive, and more cost-effective treatment strategy. However, a fundamental shortcoming of bioremediation is that the organisms used for this purpose may be unable to thrive in adverse and unfavorable environmental conditions, as well as the potential inefficiency of the process [8,9].

Enzymatic bioremediation is an emerging method of supplementing bio-treatment techniques. The class of enzyme that is most commonly used for bioremediation purposes is the “oxidoreductase” class of enzymes, which includes oxygenases, monooxygenases, dioxygenases, laccases, and peroxidases. These enzymes carry out redox reactions on a relatively wide range of substrates, including polycyclic aromatic hydrocarbons (PAHs), polynitrated aromatic compounds, pesticides, bleach-plant effluents, synthetic dyes, polymers, and various emerging pollutants [10,11,12,13]. Within the oxidoreductase family of enzymes, peroxidases such as lignin peroxidase (LiP), manganese-dependent peroxidase (MnP), versatile peroxidase (VP), soybean peroxidase (SBP), and chloroperoxidase (CPO) have been extensively studied due to their high potential for the degradation of various organic compounds [12,14,15]. It has been postulated that these enzymes could oxidize organic compounds through the generation of reactive free radicals, leading to the formation of lower molecular weight compounds and eventually mineralizing the pollutant. It is worth mentioning that some of the potential shortcomings of using enzymes for large-scale bioremediation purposes are their relatively high costs as well as the lack of the reusability of the enzymes and their stabilities during the remediation processes. However, most of these issues can be ameliorated by employing molecular biology approaches for the microbial recombinant expression of wild-type as well as mutated forms of these enzymes, which can then be immobilized on various supports, such as beads or membranes, to increase their stabilities as well as reusability [16]. The fungus *Caldariomyces fumago* secretes heme-containing CPO, also known as chloride:hydrogen-peroxide oxidoreductase. In addition to catalyzing halogenation reactions, CPO also exhibits peroxidase, catalase, and cytochrome P450-like activities. Although CPO shares common features with other heme-containing enzymes, its structure is unique, being composed of a tertiary assembly consisting primarily of eight helical segments. Our research group and others have used this enzyme for the efficient and rapid degradation of various organic pollutants [17,18,19,20,21]. Although a tremendous amount of research has been published on the use of AOPs [22,23,24] and enzymatic [18,19,25,26] approaches to the degradation of organic pollutants, including some “combination approaches” [27,28,29,30,31], to date there have been no detailed studies published that compare the degradation of a specific organic compound by these two different methods. Many questions remain about the pollutant degradation pathways, the types of intermediates generated, and the residual toxicity of the pollutants when using these two very different methods. The ultraviolet (UV) + H_2_O_2_ AOP approach relies on the pollutant molecules reacting with the hydroxyl radicals generated upon the photolysis of H_2_O_2_, whereas the CPO enzyme method relies on the enzyme heme-iron oxyradical. Therefore, it is expected that there may be significant differences in the intermediates produced during the two processes.

The aim of present study was to compare the degradation of a thiazole compound (thioflavin T, ThT) when using the classical UV + H_2_O_2_ AOP method and the CPO + H_2_O_2_ based approach. ThT can prove to be a useful model for pollution remediation studies, as its thiazole core is a common feature in some of the important emerging pollutants, such as 2-mercaptobenzothiazole (a plasticizer/vulcanizing agent), Thiabendazole and Tricyclazole (fungicidal drugs), and Meloxicam, (a non-steroidal anti-inflammatory drug). Alarmingly, all of these and other thiazole derivatives have been detected in various aquatic environments [32,33]. Moreover, as ThT is a colored compound, colorimetric changes during degradation can be monitored easily and efficiently using spectrophotometry. This study also carried out an liquid chromatography-mass spectrometry-mass spectrometry (LC-MS-MS)-based investigation to allow for a comparison of the mechanistic degradation pathways of the two remediation methods. Finally, phytotoxicity analyses were carried out to measure the residual toxicity of ThT after treatment with the AOP and CPO-based systems.

## 2. Results and Discussion

### 2.1. Degradation of ThT by UV + H_2_O_2_

**Figure 1:**
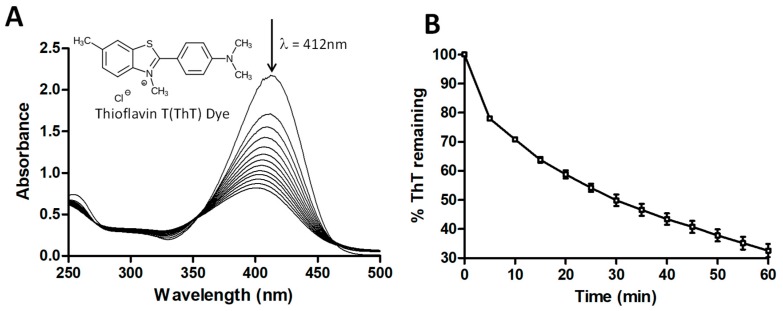
Thioflavin T (ThT) dye degradation by a UV + H_2_O_2_ advanced oxidation process. (**A**) ultraviolet/visible (UV/Vis) absorbance spectra for ThT degradation by UV + H_2_O_2_. Concentration of ThT dye = 25 ppm, pH = 2, concentration of H_2_O_2_ = 1 mM. The UV/Vis scans were taken every 5 min; (**B**) Percentage of ThT remaining (decrease in absorbance at 412 nm) after treatment with UV + H_2_O_2_. Concentration of ThT dye = 25 ppm, pH = 2, concentration of H_2_O_2_ = 1 mM. Data shown is the average of triplicate measurements (± standard deviation (SD)).

In the present work, we initially investigated the H_2_O_2_-assisted photochemical oxidation of ThT. Figure 1 shows the chemical structure as well as the absorption spectrum of ThT. Also shown is the major peak in the yellow visible region of the ThT absorption spectrum (λ_max_ = 412 nm). The exposure of the ThT solution to UV + H_2_O_2_ caused an immediate and gradual decrease in the intensity of λ_max_, indicating that new compounds had formed. Figure 1B shows the degradation of λ_max_ as a function of time, and it can be seen that about 70% of the compound had degraded after 60 min of this AOP treatment. No ThT degradation was observed in the presence of UV light or H_2_O_2_ alone. The degradation of ThT was attributed to the hydroxyl radicals (OH^•^) produced from H_2_O_2_ when exposed to UV radiation. Hydroxyl radicals are known to be strong oxidizing agents that can react with ThT molecules to produce intermediates that are responsible for decoloring/degradation of the original solution [34,35]. A simplified reaction scheme for this process is outlined below:

H_2_O_2_ + *hv* → 2 OH^•^ (1)

ThT + OH^•^ → degraded products (2)

The results obtained here are consistent with numerous studies by our group and others, which show that UV + H_2_O_2_ can readily degrade an array of organic compounds [36,37].

### 2.2. Degradation of ThT by CPO + H_2_O_2_

In order to compare the UV + H_2_O_2_-induced degradation of ThT with an enzymatic approach, we used the well-known peroxidase CPO to degrade ThT.

**Figure 2:**
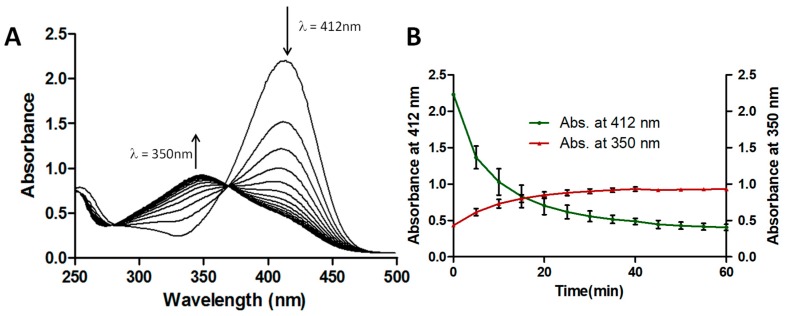
ThT dye degradation by Chloroperoxidase (CPO) + H_2_O_2_. (**A**) UV/Vis absorbance spectra for ThT dye degradation by CPO + H_2_O_2_. Concentration of ThT dye = 25 ppm, pH = 2, concentration of H_2_O_2_ = 1 mM, concentration of CPO = 10 nM. The UV/Vis scans were taken every 5 min; (**B**) Changes in absorbances at 412 nm and 350 nm of ThT dye after treatment with CPO + H_2_O_2_. Concentration of ThT dye = 25 ppm, pH = 2, concentration of H_2_O_2_ = 1 mM, concentration of CPO = 10 nM. Data shown is the average of triplicate measurements (± SD).

Figure 2 shows the absorbance spectra of ThT when exposed to CPO + H_2_O_2_ as a function of time. As was seen for the UV + H_2_O_2_ degradation of ThT in Figure 1, the ThT started to degrade immediately upon exposure to CPO + H_2_O_2_, as evidenced by the decrease in the absorbance at 412 nm. Interestingly, as the peak at 412 nm decreased in intensity, a new peak with λ_max_ = 350 nm appeared, with the intensity of this peak increasing over time. This is shown more clearly in Figure 2B. The increase in the absorbance this peak suggests that a new compound was being generated upon the treatment of ThT with CPO + H_2_O_2_, something that was not seen in the case of the UV + H_2_O_2_ treatment of ThT. This observation suggests that the UV + H_2_O_2_ and CPO + H_2_O_2_ processes likely have different effects on ThT.

### 2.3. Analysis of Product Formation Using LC-MS

**Figure 3:**
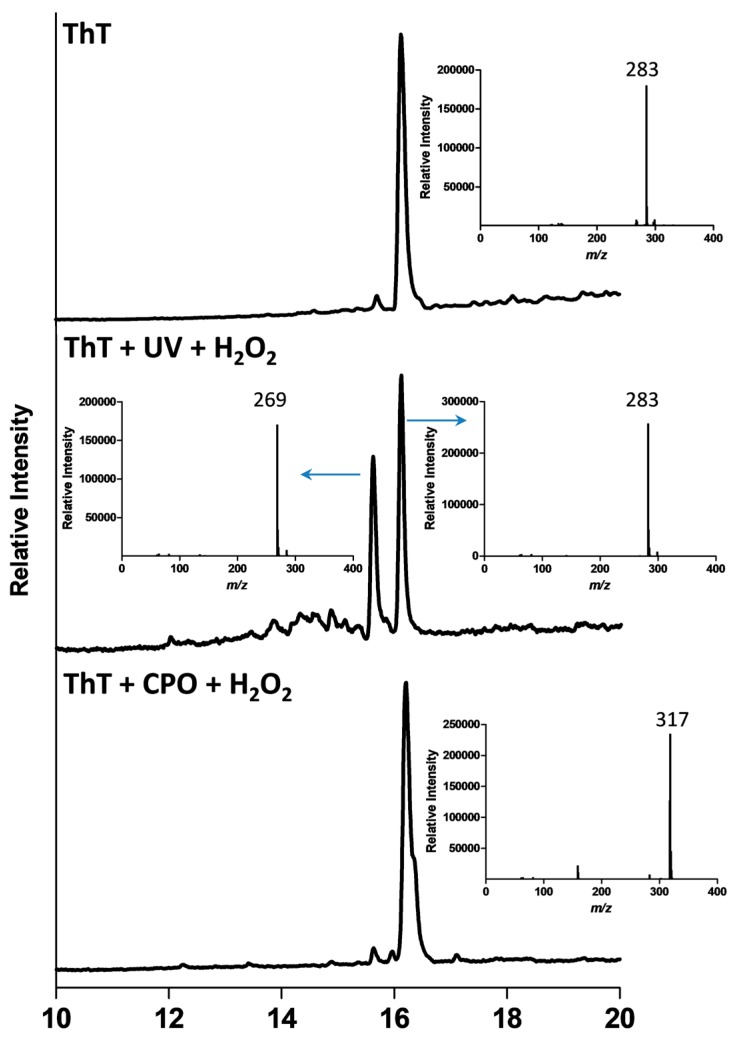
Liquid chromatography-mass spectroscopy (LC-MS) chromatogram and mass spectrometry-mass spectrometry (MS-MS) analyses of ThT degraded by UV + H_2_O_2_ and CPO + H_2_O_2_ processes. The conditions for UV + H_2_O_2_ degradation were: concentration of ThT = 25 ppm, pH = 2, concentration of H_2_O_2_ = 1 mM, and for CPO + H_2_O_2_ degradation: concentration of ThT dye = 25 ppm, pH = 2, concentration of H_2_O_2_ = 1 mM, concentration of CPO = 10 nM.

Since the UV/Vis spectroscopic data for degradation of ThT by UV + H_2_O_2_ and CPO + H_2_O_2_ suggested that different degradative pathways might be operating in the two remediation methods, we wanted to confirm this using liquid chromatography-mass spectroscopy (LC-MS). Figure 3 shows the LC-MS chromatograms of ThT, ThT exposed to UV + H_2_O_2_, and ThT treated with CPO + H_2_O_2_. Additionally, the insets show the mass spectra of the major peaks in the three samples. The LC-MS analysis of the neat ThT dye shows a single major peak at a retention time of 16.11 min, with *m*/*z* = 283 (inset). This molecular weight (MW) is in agreement with the loss of the chloride counter ion from the molecular weight of the ThT dye (MW = 318.86 Da), which would be expected as the LC-MS system was operating in the positive mode. The LC-MS analysis of the ThT + UV + H_2_O_2_ sample shows a much smaller ThT peak (retention time = 16.11 min), which is consistent with the degradation of ThT. However, another major peak can also be seen at a retention time of 15.64 min. The mass spectrum of this new product peak (inset) shows it to have an *m*/*z* value of 269. In addition to this major product peak, several other smaller peaks can also be seen at the 14–16 min range. The LC-MS analysis of the ThT dye treated with CPO + H_2_O_2_ shows a very different LC profile, with the major peak having a retention time of 16.19 min. The mass spectrum of this peak shows it to have an *m*/*z* value of 317, indicating the presence of a compound that is very different from both the original ThT (*m*/*z* = 283) and the major intermediate produced during the UV/H_2_O_2_ AOP degradation of ThT (*m*/*z* = 269). In addition to this *m*/*z* = 317 peak, additional minor peaks can also be seen in the ThT + CPO + H_2_O_2_ sample.

**Figure 4:**
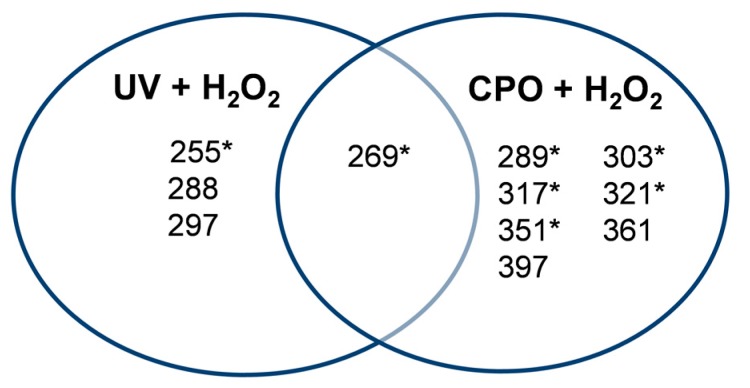
Summary of the intermediates produced upon ThT degradation by UV + H_2_O_2_ and CPO + H_2_O_2_ processes. The asterisk (*) indicate intermediates whose structures are shown in Figure 6A,B.

Detailed analyses of all the peaks detected after the UV + H_2_O_2_ and CPO + H_2_O_2_ treatments of ThT are shown in Table 1. As can be seen from this table, the AOP-mediated degradation of ThT (ThT + UV + H_2_O_2_) generated four intermediates with *m*/*z* values of 297, 288, 269, and 255, with the *m*/*z* = 269 peak being the most prominent intermediate (based on peak area). Interestingly, the intermediates produced during the enzymatic treatment of ThT (ThT + CPO + H_2_O_2_) were very different, with *m*/*z* values of 397, 361, 351, 321, 317, 303, 289, and 269. It is worth noticing that except for the *m*/*z* = 269 species, the two different treatments produced completely different intermediates. This interesting and novel finding is also graphically represented in Figure 4.

### 2.4. Mechanistic Studies 

**Figure 5:**
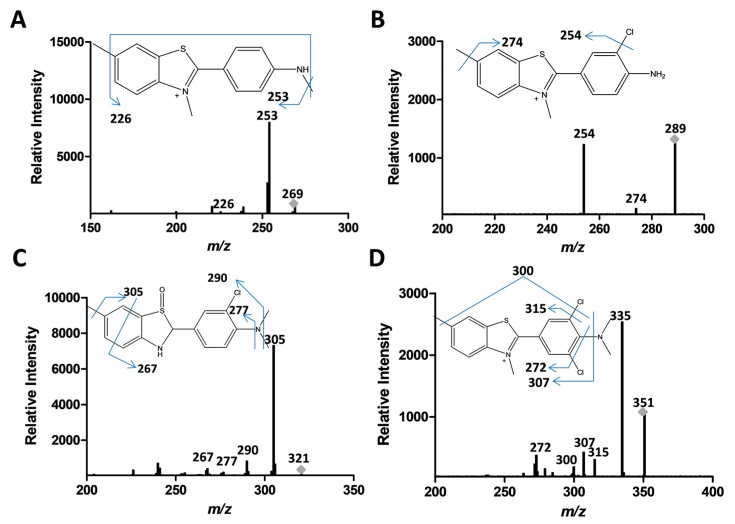
Tandem mass spectrometry fragmentation analyses of proposed intermediates produced after ThT degradation by UV + H_2_O_2_ (*m*/*z* of 269, panel (**A**)) or CPO + H_2_O_2_ as shown in panels (**A**,**B**) (*m*/*z* of 289); (**C**) (*m*/*z* of 321) and (**D**) (*m*/*z* of 351).

An attempt was made to propose plausible structures for the various different intermediates generated in the two different treatments by using tandem mass spectrometry-mass spectrometry (MS-MS) data. Indeed, as can be seen in Figure 5, we were able to propose structures for seven of the intermediates produced, with the relevant peaks being indicated by asterisks (*) in Figure 4. Based on the structure of the intermediates, we were able to develop plausible schemes for ThT breakdown during the UV + H_2_O_2_ and CPO + H_2_O_2_ remediation processes. In the UV + H_2_O_2_ process, the main mechanism involves the formation of OH^•^ radicals by the UV-mediated homolysis of hydrogen peroxide. These reactive OH^•^ radicals attack ThT, leading to the stepwise demethylation of the tertiary amine and resulting in the formation of intermediates with *m*/*z* values of 269. Subsequent demethylation produces the *m*/*z* = 255 compound. This demethylation mechanism has been previously reported by our group as well as others [38,39,40]. The decrease in the retention times of these intermediates (15.64 min and 15.8 min) on the reverse-phase column when compared to ThT (16.11 min) reflects their increased polarities. Other, more polar intermediates with *m*/*z* values of 297 and 288 and retention times of 15.11 and 14.35 min, respectively, were also produced during the UV + H_2_O_2_ treatment of ThT.

Unlike the UV-induced photolysis of H_2_O_2_, which generates reactive hydroxyl radicals, the CPO + H_2_O_2_ system entails a different mechanism. It is well-known that heme peroxidases, such as CPO, can react with H_2_O_2_ to generate an enzymatic iron oxyradical called Compound I, which can react with organic substrates to be converted to the Compound II form of the enzyme and an organic radical. The Compound II form of the enzyme can react with another organic substrate to create another organic radical molecule and regenerate the resting form of the enzyme [9], as shown below:

Peroxidase + H_2_O_2_ → Compound I + H_2_O (3)

Compound I + SH → Compound II + S^•^ (4)

Compound II + SH → Peroxidase + S^•^ + H_2_O (5)

where SH indicates a generic substrate.

Although the above peroxidase reaction cycle does not explicitly show the generation of hydroxyl radicals, it is possible that OH radicals may also be produced in this case, as CPO is known to cleave the peroxide O–O bond through a glutamic acid residue present in its active site [41].

Besides the well-known H_2_O_2_-peroxidase cycle described above, CPO is also known to catalyze chlorination of organic compounds [18,42], as shown below:

R–H + Cl^−^ + H_2_O_2_ + H^+^ → R–Cl + 2 H_2_O (6)

**Figure 6:**
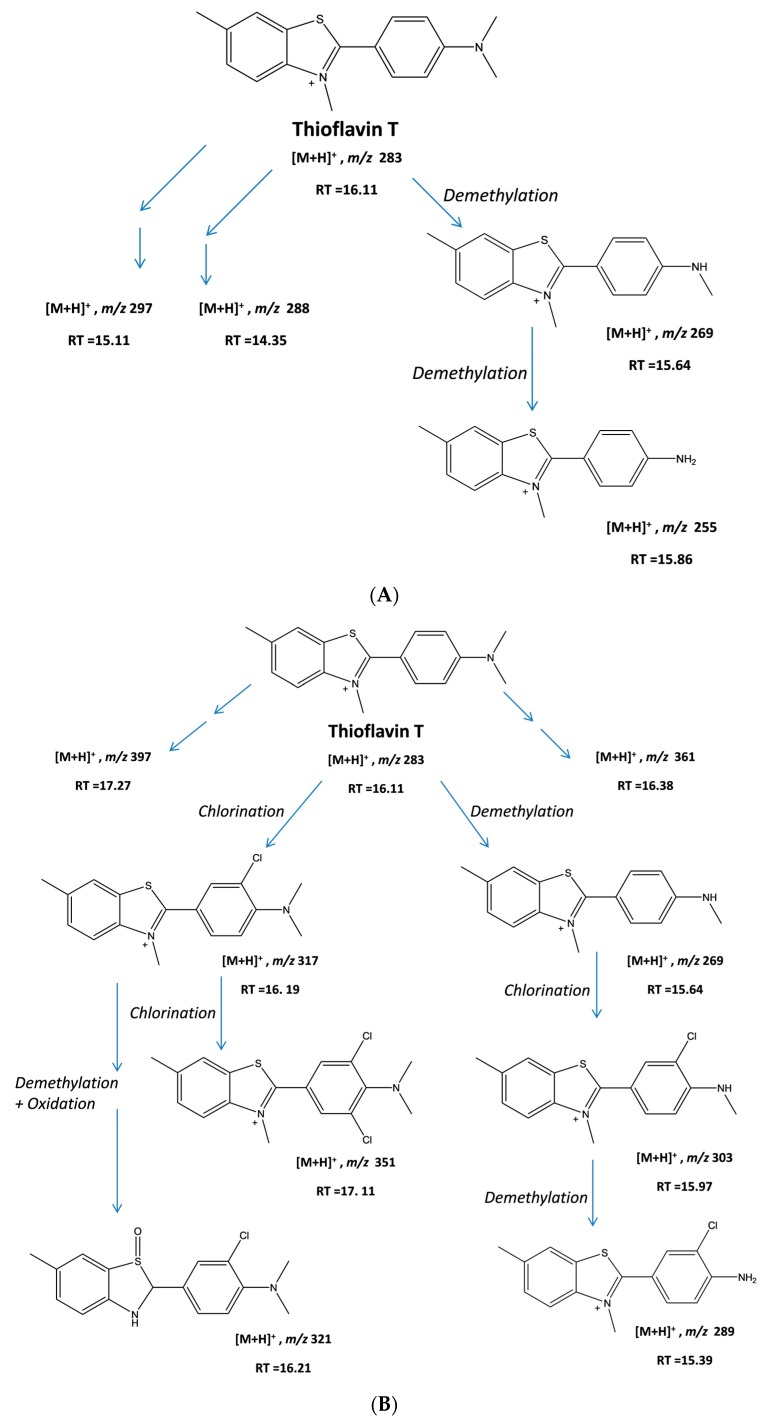
(**A**) Proposed structures of some of the intermediates generated during the UV + H_2_O_2_-based degradation of ThT dye; (**B**) Proposed structures of some of the intermediates generated during the CPO + H_2_O_2_-based degradation of ThT dye. RT: retention time.

In fact, based on the MS-MS data, our proposed degradation scheme suggests that the action of CPO on ThT in the presence of H_2_O_2_ can occur through two overlapping pathways, with the first being the chlorination of ThT, and the second being the production of OH^•^ that can react with ThT and its chlorinated products (Figure 6B). A comparison of the abundance of the proposed species (Table 1) as determined by LC-MS-MS suggests that the chlorination pathway (317 *m*/*z* species) was at least twenty times more active than the demethylation pathway (269 *m*/*z* species). A second chlorination also occurs, yielding a compound with an *m*/*z* value of 351 (Figure 5D). The mono-chlorinated ThT could also undergo stepwise demethylation, yielding structures with *m*/*z* values of 303 and 289. An intermediate with *m*/*z* = 321 was also observed, which corresponds to the addition of an OH (oxidation) to the demethylated form of chlorinated ThT, as seen in Figure 6B.

The generation of chlorinated products of ThT observed during the CPO + H_2_O_2_ treatment of ThT was not completely unexpected, as ThT contains a chloride counter ion and it is well-established that CPO, in the presence of halide ions can lead to the halogenation of various organic substrates [43]. In fact, we have previously published that in contrast to ThT, a different but related thiazole compound (2-mercaptobenzothiazole), which did not contain a chloride counterion, did not produce any chlorinated products [18]. However, it is expected that most industrial waste streams would have relatively high concentrations of various ions, including halides [44], and hence the results presented here could be valuable and relevant in real life remediation situations.

### 2.5. Toxicity Studies

**Figure 7:**
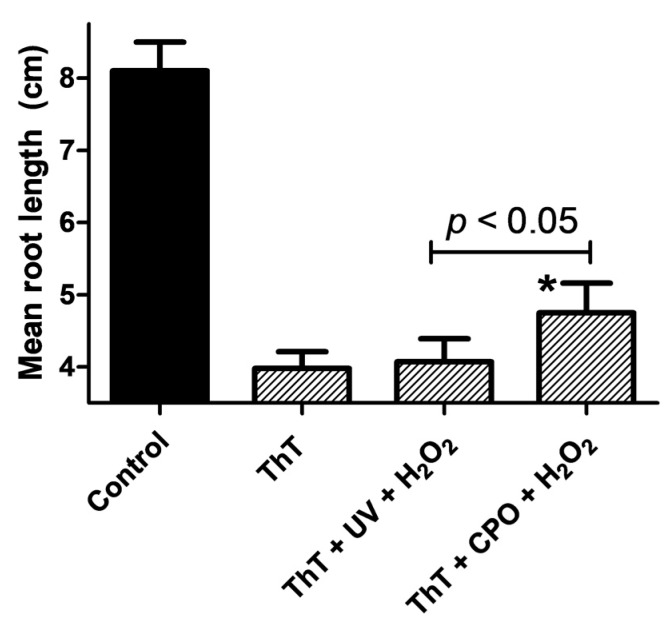
ThT dye toxicity on *Lactuca sativa* seeds, as measured by the mean root lengths (cm) of seeds after treatment with ThT dye (25 ppm), ThT dye treated by UV + H_2_O_2_, or ThT dye treated by CPO + H_2_O_2_. Statistical analyses were performed using an unpaired *t*-test (*n* = 40); asterisk (*) denotes a significant difference (*p* < 0.05).

The toxicity of degradation products should be analyzed where possible, as they can often be more toxic than the parent compound [45]. Therefore, we carried out phytotoxicity studies using *Lactuca sativa* seeds by exposing them to AOP-remediated ThT and enzymatically treated ThT solutions. The germination of *Lactuca sativa L. var. Buttercrunch* seeds is a standard protocol for assessing toxicity in water and soil matrices, and is recommended for bioassays by the U.S. Environmental Protection Agency, the Food and Drug Administration, and the Organization for Economic Cooperation and Development [46]. Figure 7 shows the results of the toxicity studies, in which the root lengths of *L. sativa* seeds exposed to distilled water (control), neat ThT, ThT treated with UV + H_2_O_2_, or ThT treated with CPO + H_2_O_2_ were measured. As can be seen from the figure, ThT had a significant phytotoxic effect on the seeds, causing a dramatic and significant decrease in the mean root length. A significant inhibition of root lengths was also observed in the ThT + UV + H_2_O_2_ and ThT + CPO + H_2_O_2_ samples (Figure 7). However, a *t*-test analysis showed that the ThT + CPO + H_2_O_2_ sample was significantly (*p* < 0.05) less toxic than the ThT + UV + H_2_O_2_ sample. This result was quite unexpected, and suggests that one or more of the intermediates generated during the UV + H_2_O_2_ treatment of ThT could be toxic. Alternatively, the fact that the ThT + CPO + H_2_O_2_ sample still exhibited significant phytotoxicity could be explained by the fact that this sample still contained large amounts of the toxic undegraded ThT dye. Further studies will need to be carried out to allow the nature of the phytotoxicity of the ThT + UV + H_2_O_2_ sample to be understood; however, the present data shows that the UV + H_2_O_2_ and CPO + H_2_O_2_ treatments of ThT produce different intermediates that could have differing toxicities: an observation that has not been published earlier.

## 3. Materials and Methods

### 3.1. Reagents

The thiazole compound thioflavin T (whose molecular formula is C_17_H_19_ClN_2_S and whose formula weight is 318.86 g·mol^−1^) was purchased from AnaSpec (Fremont, CA, USA). Hydrogen peroxide (30% *w*/*v*) and LC-MS grade solvents, such as formic acid and acetonitrile, were purchased from Sigma-Aldrich (St. Louis, MO, USA). All of the experiments were carried out in 50 mM citrate buffer, pH 2. CPO with a specific activity of 1296 IU/mg (17 mg/mL, 405 µM) was purchased from Bio-Research Products (North Liberty, IA, USA).

### 3.2. Thioflavin T Decolorization

For the photolytic treatment of ThT using UV + H_2_O_2_, 1 mM H_2_O_2_ was added to ThT samples in 50 mM citrate buffer (pH 2), which were then irradiated with a UV lamp (UVGL-58, J-129, Upland, NJ, USA) from a distance of 1.5 cm. The instrument had a UV power output of 6 W and was selectively used in the 254 nm output mode for these studies. Under these conditions, no significant warming up of the irradiated solution was observed.

For the enzymatic treatment of ThT using CPO + H_2_O_2_, experiments were carried out as previously described [18]. Briefly, ThT and H_2_O_2_ (1 mM) in 50 mM citrate buffer (pH 2) was exposed to CPO (10 nM), and the changes in the full ThT spectrum were monitored.

In both the enzymatic and the photolytic studies, spectra were collected in the range of 200–800 nm using a Carry 60 Spectrophotometer (Agilent Technologies, Santa Clara, CA, USA), with a path length of 1 cm (4 mL quartz cuvette) and 412 nm was used as λ_max_.

### 3.3. LC-MS and MS-MS Analyses

The materials produced after the enzymatic and the photolytic treatments were analyzed using LC-MS, as previously described [18,19]. Briefly, all ThT samples were filtered using a 0.45 µm CA syringe filter prior to injection. The LC-MS was fitted with a ZORBAX Eclipse Plus C18 column (Agilent Technologies, Santa Clara, CA, USA) with a particle size of 1.8 µm, an inner diameter of 2.1 mm, and a length of 50 mm. The column was maintained at 35 °C, and a constant flow rate of 0.2 mL/min was maintained. The column was coupled to a 6420 Triple Quad LC-MS System detector (Agilent Technologies). Two mobile phases were used: A is water containing 0.1% formic acid, and B is 100% acetonitrile. The LC method was set as follows: 5 min of 100% A, followed by a 0–100% gradient of B from 5–20 min, then 5 min of 100% B after the gradient, and finally 5 min of 100% A. The electrospray ionization source in the LC-MS system was in positive polarity mode, the capillary voltage was set at 4000 V, the nebulizer pressure was maintained at 45 psi, the drying gas (N_2_) flow was 11 L/min, and the drying temperature was set at 325 °C. The mass range monitored for all of the runs was between 50 and 1000 Da. In the tandem MS experiments using the product ion mode, nitrogen gas was used for fragmentation and different collision energies were used.

### 3.4. Phytotoxicity Assay

The toxicity of ThT before and after the UV + H_2_O_2_ and CPO + H_2_O_2_ treatments was measured using the lettuce seed growth inhibition assay, similar to a previously described method but with slight modifications [19]. Briefly, 20 *Lactuca* *sativa* seeds were placed on sterilized Whatman filter paper, No. 3, in a Petri dish and saturated with 4 mL of the samples. The petri dishes were incubated for 5 days in a humidified chamber at 25 ± 2 °C. Distilled water was used as a negative control and ThT was used as a positive control, with each sample being tested in duplicate. The effects of the original dye and the dye samples degraded by UV + H_2_O_2_ and CPO + H_2_O_2_ were examined by measuring the lengths of the roots of the germinated seeds. Statistical analyses were conducted for each group of treated seeds (*n* = 40). The data were analyzed via unpaired *t*-tests. Data are reported as group mean ± standard deviation, and significance for all statistical comparisons was set at *p* < 0.05.

## 4. Conclusions

To the best of our knowledge, this is the first study to present a systematic comparison of the use of two different remediation methods to treat a toxic organic pollutant. We showed that treating ThT with UV + H_2_O_2_ and CPO + H_2_O_2_ produced very different degradation products, with only one common intermediate. This suggested that different organic pollutant degradation schemes were involved in these two remediation approaches, and we attempted to elucidate these mechanisms. Additionally, we showed that the AOP (UV + H_2_O_2_) and enzymatic (CPO + H_2_O_2_) treated ThT solutions had significantly different toxicities for *L. sativa* seeds. The unexpected and intriguing data presented here also highlights the need for a better understanding of different remediation approaches and for additional research into such comparative remediation studies.

## Figures and Tables

**Table 1:** Peak area of intermediates produced during the degradation of ThT by UV + H_2_O_2_ and CPO + H_2_O_2_ processes.

Retention Time (min)	ThT + UV + H_2_O_2_		ThT + CPO + H_2_O_2_	
	*m/z*	Peak Area	*m/z*	Peak Area
14.35	288	24,398	-	-
14.86	255	161,335	-	-
15.11	297	70,562	-	-
15.39	-	-	289	27,169
15.64	269	1,216,238	269	300,854
15.97	-	-	303	190,007
16.11	283	1,789,254	283	249,499
16.19	-	-	317	6,147,872
16.21	-	-	321	138,735
16.38	-	-	361	639,968
17.11	-	-	351	81,812
17.27	-	-	397	9,472
